# Procyanidin B2 3″-*O*-gallate Isolated from *Reynoutria elliptica* Prevents Glutamate-Induced HT22 Cell Death by Blocking the Accumulation of Intracellular Reactive Oxygen Species

**DOI:** 10.3390/biom9090412

**Published:** 2019-08-26

**Authors:** Ji Hoon Song, Sil Kim, Jae Sik Yu, Do Hwi Park, Song-Yi Kim, Ki Sung Kang, Sullim Lee, Ki Hyun Kim

**Affiliations:** 1 Jeju Institute of Korean Medicine, Jeju 63309, Korea; 2 School of Pharmacy, Sungkyunkwan University, Suwon 16419, Korea; 3 College of Korean Medicine, Gachon University, Seongnam 13120, Korea; 4 Department of Anatomy and Acupoint, College of Korean Medicine, Gachon University, Seongnam 13120, Korea; 5 Department of Life Science, College of Bio-Nano Technology, Gachon University, Seongnam 13120, Korea

**Keywords:** *Reynoutria elliptica*, neuroprotective effects, procyanidin B2 3″-*O*-gallate, HT22, apoptosis, mitogen-activated protein kinases

## Abstract

In this study, we examined the neuroprotective effects of MeOH extract and bioactive compounds obtained from *Reynoutria elliptica* seeds using HT22 cells from the murine hippocampal cell line as its underlying molecular basis, which has not yet been elucidated. Our study showed that the MeOH extract of *R. elliptica* seeds strongly protected HT22 cells from glutamate toxicity. To clarify the responsible compound for the neuroprotective effects, we took an interest in procyanidins of *R. elliptica* since procyanidins are known to exhibit high structural diversity and neuroprotective activity. To isolate the procyanidins efficiently, a phytochemical investigation of the MeOH extract from *R. elliptica* seeds using the LC/MS-guided isolation approach was applied, and procyanidin B2 3″-*O*-gallate (**1**) was successfully isolated. The structure of **1** was elucidated by analyzing the nuclear magnetic resonance spectroscopic data and LC/MS analysis. The neuroprotective activities of **1** were thoroughly examined using HT22 cells. Compound **1** exhibited a strong antioxidant efficacy and blocked glutamate-mediated increase in the reactive oxygen species (ROS) accumulation. Furthermore, compound **1** significantly inhibited the phosphorylation of extracellular signal-regulated kinase, p38, and c-Jun N-terminal kinase, which were increased by glutamate. These findings prove that the extract of *R. elliptica* seeds containing procyanidin B2 3″-O-gallate, which is a strong neuroprotective component, can be used as a functional food forattenuating and regulating neurological disorders.

## 1. Introduction

Neurological disorders are major pathological conditions caused by dysfunctions in the brain and are associated with neuronal cell death in specific regions of the brain. Extensive in vitro and in vivo studies have been contributed to define the mechanism of neuronal cell death in neurological disorders [1]. Nonetheless, the exact pathogenesis of these neurological disorders is not yet clearly understood, which is one of the obstacles for using current drugs in the treatment of neurological disorders [2]. Furthermore, currently available drugs have multiple adverse effects. Therefore, new strategies are required to discover drugs that overcome these issues. Extracts and bioactive compounds from natural sources are possible neuroprotective candidates for various neurological disorders. Natural products possess various biological potentials including antioxidant, anti-inflammatory, and anti-apoptotic activities [3]. These natural products and nutraceuticals have been shown previously to prevent neuronal cell death, enhance memory, and improve cognitive capacity using in vitro and in vivo models for neurological disorders [4]. Therefore, finding natural products with protective properties against neuronal cell death is important for regulating and treating neurological disorders.

*Reynoutria elliptica* (Koidz.) Migo, known as a member of the Polygonaceae family, grows widely in Korea, China, Japan, and North America. In Korea, the root and rhizome of *R. elliptica* have been used not only as a functional food for laxative, emmenagogue, diuretic, and antitussive but also treatment for cystitis, suppurative dermatitis, gonorrhea, and favus [5,6,7]. Furthermore, in China, this species called “Hu-zhang” is stated in the Chinese Pharmacopoeia. The species has also been used in sweet and sour beverages and boiled down with licorice. Currently, the extracts of the roots and rhizomes are orally or topically administered in various forms, such as powders, granules, liquid, and tinctures. They are also used as a natural source of resveratrol, which is a well-known dietary supplement. 

Previous studies have demonstrated that the *R. elliptica* root displays various pharmacological activities including a neuraminidase inhibitory effect for preventing and treating influenza infections [8], anti-inflammatory [6], antidiabetic [9], anticancer, and antimicrobial activities [10], and a neuroprotective effect on retinal ganglion cells [5]. Previous chemical studies on *R. elliptica* have shown the presence of various compounds including quinones, stilbenes, flavonoids, coumarins, lignans [10], and procyanidins [11,12]. Among these substances, we have taken an interest in procyanidins, which have various biological properties, including antibacterial [13], antiatherosclerotic, antilipidemic [14], antioxidant [15,16], anti-inflammatory [17,18], and neuroprotective activities [19]. However, the seeds of *R. elliptica* have never been used as a functional food, unlike the roots and rhizomes. Based on our knowledge, a chemical investigation of the seeds has not been conducted to date. Moreover, the neuroprotective effect of *R. elliptica* seeds on brain cells has not yet been examined. Thus, the neuroprotective activity and molecular and cellular mechanisms underlying this effect must be elucidated.

As part of our ongoing projects to screen Korean natural resources possessing pharmacological capability and its responsible compounds [20,21,22,23,24,25,26,27,28,29], we assessed the neuroprotective roles of the MeOH extract of *R. elliptica* seeds in immortalized murine hippocampal neuronal cell line, HT22 cells, which is part of the murine hippocampal cell line. We further characterized the compounds responsible for its neuroprotective effects as procyanidins of *R. elliptica*. Using an liquid chromatography (LC)/mass spectrometry (MS)-guided isolation technique, procyanidin B2 3″-*O*-gallate (**1**), which is a promising constituent that is responsible for the neuroprotective effect, was isolated, and the structure of the isolate (**1**) was evaluated using the nuclear magnetic resonance (NMR) spectroscopic and LC/MS analysis. To the best of our knowledge, neuroprotective effects of *R. elliptica* seed extracts against glutamate-induced neurotoxicity has not been studied yet and researchers have not identified its bioactive constituents as of yet. In this paper, we report the neuroprotective effects and basic underlying mechanism of extracts and the bioactive constituent derived from *R. elliptica* seeds using HT22 cells.

## 2. Materials and Methods

### 2.1. Plant Material

The seeds of *R. elliptica* were gathered from the Hantaek botanical garden (Yongin, Korea) in October 2001. A voucher specimen (RE-2001-10) was authenticated by the plant managing director of Hantaek botanical garden (Jung Hwa Kang) and stored at the herbarium of the Korea Plant Extract Bank, Cheongju, Republic of Korea.

### 2.2. Extraction and Isolation

Dried seeds of *R. elliptica* (110 g) were extracted three times with 99.9% MeOH (200 mL × 3 days) at room temperature and then the extracts were filtered. The filtrates were combined and concentrated *in vacuo*, which yielded a crude MeOH extract (8.3 g). The details for isolation of compound **1** from the MeOH extract using the LC/MS-guided isolation method are described in the Supplementary Materials.

### 2.3. Quantitative Analysis of Procyanidin B2 3″-O-gallate 

The detection of **1** was analyzed using an LC/MS, Agilent 1200 Series analytical system equipped with a photodiode array detector combined with a 6130 Series electrospray ionization (ESI) mass spectrometer (Agilent Technologies, Santa Clara, CA, USA). Calibration curves and linear regression equations were generated for the external standard, compound **1**. The quantification of **1** is described in the Supplementary Materials.

### 2.4. Cell Culture and Treatment

The HT22 cell line was maintained and grown in Dulbecco’s Modified Eagle’s medium (Corning, Manassas, VA, USA) supplemented with 10% FBS and penicillin/streptomycin (Grand Island, NY, USA). For the experiment, the cells were plated onto multi-well plates at a density of 3 × 10^4^ cells/cm^2^. After a 24-h incubation for adherence, the extract or compound was added into the cultured cells with or without 5 mM glutamate.

### 2.5. Determination of Cell Viability

Cell viability was determined by using the EZ-CyTox cell viability assay kit (Daeil Lab Service, Seoul, Korea), according to the manufacturer’s instructions. The cell viability was represented by the percentage of control cells. 

### 2.6. Determination of Antioxidant Activity and Intracellular ROS Levels

The antioxidant activity was assessed by using 2,2-diphenyl-1-picrylhydrazyl (DPPH) radical scavenging assay. We then determined the levels of intracellular ROS using 2′,7′-dichlorofluorescin diacetate (DCFDA, Sigma-Aldrich, St. Louis, MO, USA). The cells were exposed to 5 mM glutamate for 8 h and stained with DCFDA. The fluorescent intensity of dichlorofluorescin (DCF) was measured using a fluorescent microplate reader, and the representative fluorescent images were obtained using a fluorescence microscope (IX50, Olympus, Tokyo, Japan) equipped with a charge-coupled device (CCD) camera. Intracellular levels of ROS were represented as a fold-increase compared with the control cells. 

### 2.7. Determination of Apoptotic Cells

Chromatin condensation was observed after staining the cells with 10 µM Hoechst 33342 (Sigma) for 10 min. The fluorescent images were then photographed using a fluorescence microscope (IX50, Olympus) equipped with a charge-coupled device (CCD) camera. To assess apoptotic cell death, the cells were harvested and labeled with Alexa Fluor 488-conjugaged annexin V (Invitrogen, Eugene, OR, USA) and propidium iodide, respectively. The fluorescent images were then obtained using a Tali™ Image-Based Cytometer (Invitrogen).

### 2.8. Western Blot Analysis

Cells were lysed with radio-immunoprecipitation assay buffer (Cell Signaling, Danvers, MA, USA) containing freshly added protease inhibitor cocktail (Roche, Indianapolis, IH, USA), 1 mM sodium orthovanadate, and 1 mM sodium fluoride. The same amount of proteins was separated using sodium dodecyl sulfate-polyacrylamide gel electrophoresis (SDS-PAGE) and transferred to a polyvinylidene difluoride membrane (Merck Millipore, Darmstadt, Germany). After blockading the membranes with 5% skim milk to eliminate the nonspecific bindings of antibodies, the membranes were probed with primary antibodies for extracellular signal-regulated kinase (ERK), phospho-ERK, c-Jun N-terminal kinase (JNK), phospho-JNK, p38, phospho-p38, and glyceraldehyde 3-phosphate dehydrogenase (GAPDH) followed by incubating with HRP-conjugated anti-rabbit IgG (Cell Signaling). Immunoreactive bands were visualized with SuperSignal West Femto Maximum Sensitivity Substrate (Thermo Scientific, Rockford, IL, USA) and quantitatively analyzed using Image J (version 1.51j8) software.

### 2.9. Statistical Analysis

All experiments were carried out at least three times. The data were represented as the mean ± standard error of the mean (SEM). Statistical significance was determined using the Student’s *t*-test, and *p* < 0.05 was considered statistically significant. 

## 3. Results and Discussions

### 3.1. Neuroprotective Effects of the MeOH Extract from R. elliptica Seeds on Glutamate-Induced Excitotoxicity in an HT22 Cell Line

**Figure 1:**
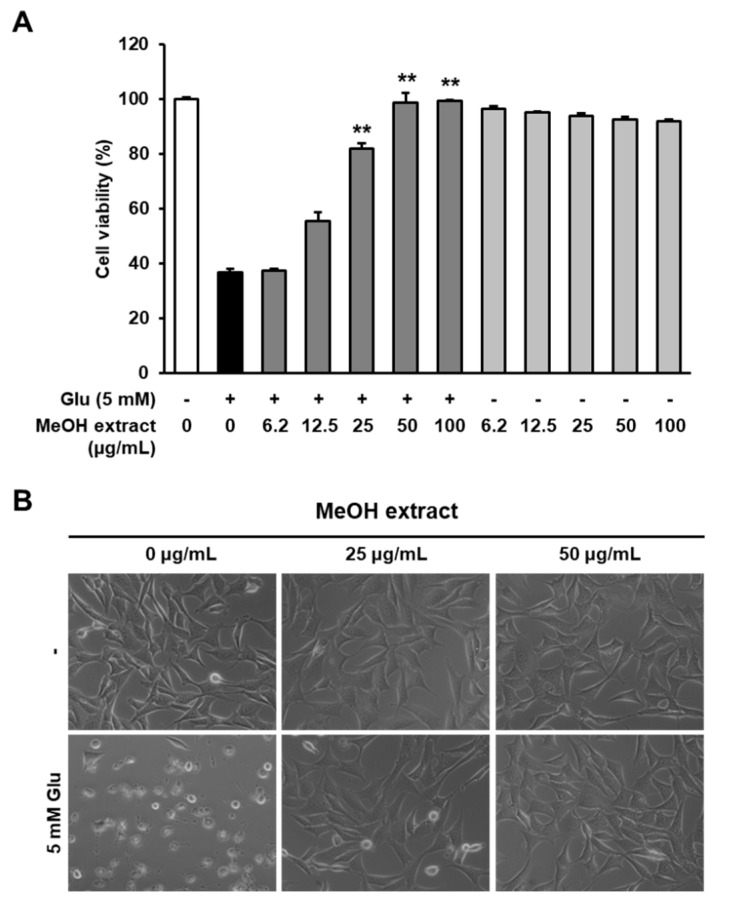
MeOH extract of *R. elliptica* seeds protect HT22 cells from glutamate-induced toxicity. (**A**) HT22 cells were exposed to the indicated concentrations of the MeOH extract of *R. elliptica* seeds with or without 5 mM glutamate (Glu) for 24 h. Bars denote the percentage of viable cells (** *p* < 0.001 compared with glutamate-treated group). (**B**) Morphological changes are represented by microscopic images (40×).

To investigate the biological effects of *R. elliptica* seeds, we prepared an MeOH extract from *R. elliptica* seeds and tested whether it protected the HT22 cells from glutamate-induced excitotoxicity. In our results, MeOH extract significantly increased the cell viability compared with glutamate-treated cells in a dose-dependent manner, and the most effective concentrations of extracts was 50 µg/mL (Figure 1A). Microscopic images also exhibited that MeOH extract completely inhibited on HT22 cell death was induced by glutamate (Figure 1B). Therefore, these results suggest that *R. elliptica* seeds are an attractable natural resource to investigate the bioactive compounds, which are contributing the neuroprotection.

### 3.2. LC/MS-Guided Isolation and Identification of the Active Compound from the MeOH Extract

**Figure 2:**
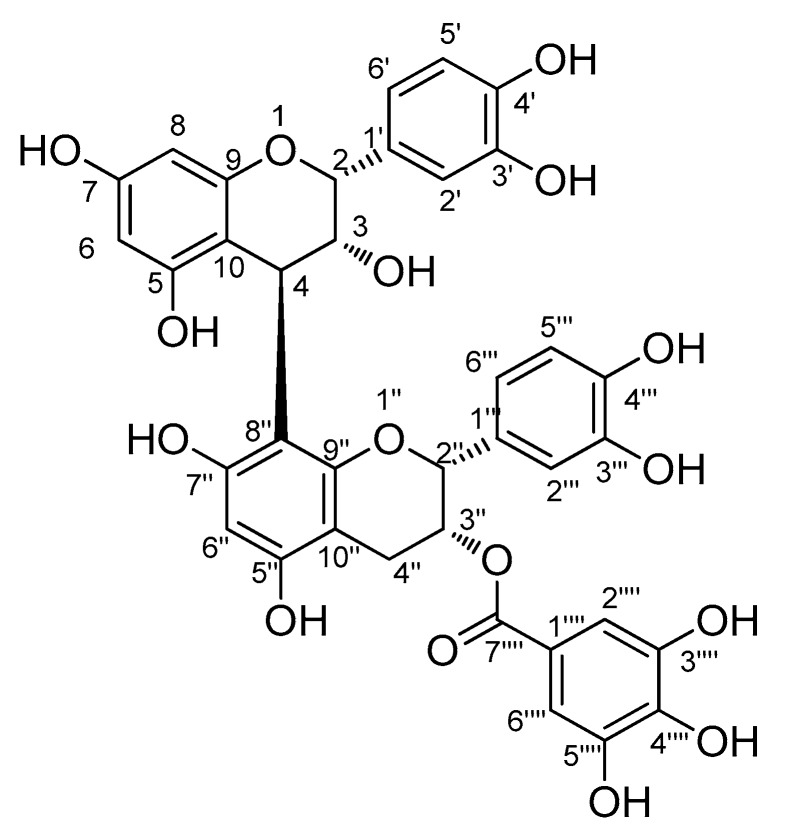
The chemical structure of **1**.

Since the MeOH extract of *R. elliptica* seeds showed a strong neuroprotective effect, we further analyzed the MeOH extract to identify the active constituent that contributed to this activity. We took an interest in procyanidins of *R. elliptica* as promising constituents since procyanidins are known to exhibit high structural diversity [11,12] and neuroprotective activity [19]. To isolate the procyanidins efficiently, the LC/MS-guided isolation approach was applied using our in-house ultraviolet (UV) library. The MeOH extract showed the existence of procyanidin dimer gallate through LC/MS analysis with a molecular ion peak at *m/z* 731 [M + H]^+^ in the positive ESI mode (Figure S1). The high selectivity and sensitivity of the LC/MS-guided isolation approach selectively shorten the analysis time and allowed time-efficient isolation of the target compound. The target compound, known as a procyanidin dimer gallate, was purified using preparative high performance liquid chromatography (HPLC) monitored by LC/MS, which led to the identification of a fraction with the desired compound. Lastly, LC/MS analysis of the F2 fraction showed signs of procyanidin dimer gallate with a molecular ion peak at *m/z* 731 [M + H]^+^ and UV absorption indicative of the procyanidin dimer (Figure S1), and it was further purified by semi-preparative HPLC in the F2 fraction. The isolated compound was identified as procyanidin B2 3″-*O*-gallate (**1**) by comparing the NMR spectroscopic (Figures S2 and S3) and physical data with previously reported values (Table S1) [30], together with LC/MS analysis (Figure 2). Quantitative analysis of **1** by LC-MS exhibited its content to be 14.30 ± 1.92 µg/mg of the MeOH extract weight.

### 3.3. Neuroprotective Efficacy of 1 Against Glutamate-Induced HT22 Cell Death

**Figure 3:**
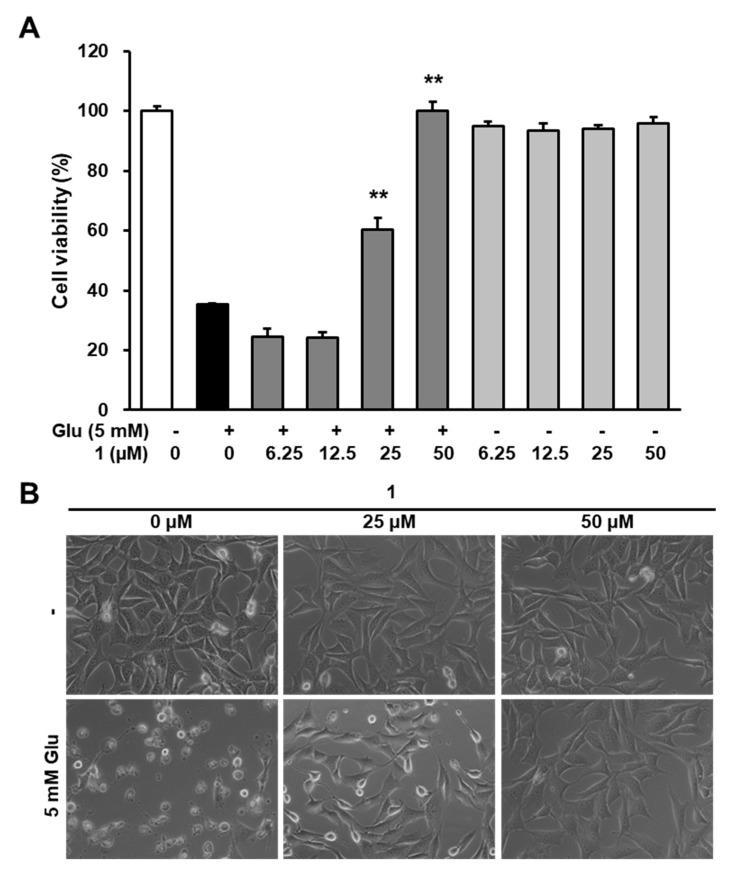
Compound **1** prevents HT22 cell death induced by glutamate. (**A**) The cells were exposed to 5 mM glutamate and the indicated concentrations of **1** for 24 h. The cell viability was determined using an EZ-CyTox cell viability assay kit. Bars denote the percentage of viable cells (** *p* < 0.001 compared with the glutamate-treated group). (**B**) Morphological changes were represented by microscopic images (40×).

Procyanidins extracted from grape seeds have shown a neuroprotective effect on an animal model of ischemic brain injury [31]. Several studies have reported that procyanidin B2 showed a strong neuroprotective effect in in vitro and in vivo models for neurological disorders [32,33]. However, the neuroprotective effect of **1** has not been studied to date. Therefore, in this study, we tested whether **1** exhibits the neuroprotective effects on the HT22 cell death induced by glutamate. Our results indicated that the presence of **1** significantly increases in cell viability compared with glutamate-treated cells (Figure 3A). Consistent with this, microscopic images also show that **1** blocked HT22 cell death induced by glutamate (Figure 3B). Thus, these results are the first to report the neuroprotective effect of procyanidin B2 3″-*O*-gallate (**1**) isolated from *R. elliptica* seeds.

### 3.4. Effects of 1 on Glutamate-Induced Reactive Oxygen Species (ROS) Accumulation

**Figure 4:**
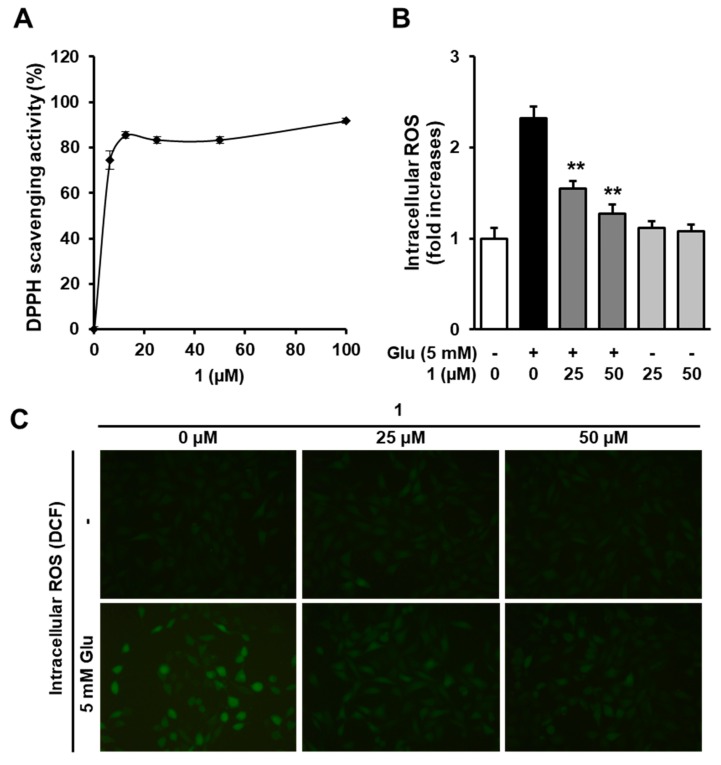
Compound **1** shows strong antioxidant activity and prevents intracellular ROS increased by glutamate. (**A**) The antioxidant activity of **1** was determined by using DPPH radical scavenging activity. (**B**) HT22 cells were exposed to 5 mM glutamate with 25 and 50 µM of **1** for 8 h and then stained with H_2_DCFDA. The fluorescence intensity of DCFDA indicating intracellular ROS was determined using a microplate reader and represented by fold increases in the control group (** *p* < 0.001 compared with the glutamate-treated group). (**C**) Fluorescent images were obtained using a fluorescence microscope (40×).

Oxidative stress, which is one of the most critical factors for neuronal cell death in the brain, is characterized by excessive accumulation of intracellular ROS due to an imbalance between ROS production and the cellular defense system [34]. Therefore, preventing oxidative stress is valuable in order to ameliorate neurons from damage. In the present study, the results indicated that **1** possessed a strong antioxidative efficacy characterized by DPPH radical scavenging activity (Figure 4A). Thus, compound **1** protects HT22 cells from glutamate toxicity. Previous studies have suggested that high concentrations of glutamate excessively increased the intracellular ROS, which resulted in oxidative stress [35,36]. Therefore, we confirmed the effect of **1** on the glutamate-mediated elevation of intracellular ROS. Quantitatively analyzed data showed that **1** significantly decreased the fluorescent intensity of DCF, which indicated the levels of intracellular ROS (Figure 4B). The fluorescent microscopic images also showed that the increase in intracellular ROS after glutamate treatment significantly decreased due to the presence of **1** (50 µM) (Figure 4C). These data suggest that **1** possesses strong neuroprotective activity when inhibiting oxidative stress triggered by glutamate in the HT22 cell.

### 3.5. Inhibitory Effect of 1 on Glutamate-Induced Phosphorylation of Mitogen-Activated Protein Kinases (MAPKs) in HT22 Cells

**Figure 5:**
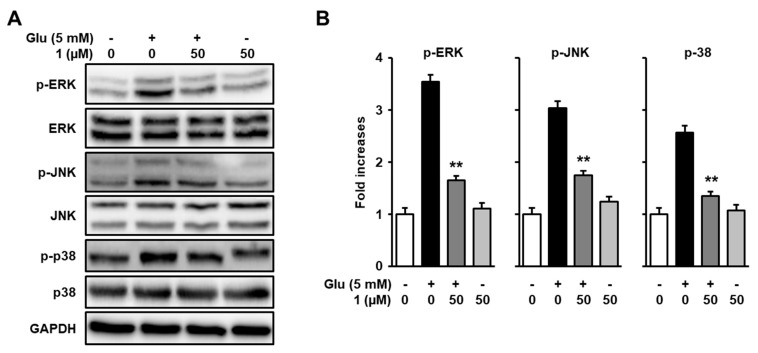
Glutamate-induced phosphorylation of MAPKs is inhibited by **1**. (**A**) HT22 cells were incubated with 5 mM glutamate and 50 µM of **1** for 8 h, which was followed by Western blot analysis. Immunoreactive bands were detected using antibodies for p-ERK, ERK, p- JNK, JNK, p-p38, p38, and GAPDH. (**B**) Bars denote the fold-increase of phosphorylation of MAPKs in the control (** *p* < 0.001 compared with glutamate-treated groups).

It has been well-known that mitogen-activated protein kinase (MAPK), which is a family of serine/threonine protein kinases, contribute to various physiological and pathological processes [37]. Previous studies suggested that the prolonged activation of MAPKs including extracellular signal–regulated kinase (ERK), c-Jun N-terminal kinase (JNK), and p38 was closely related to oxidative stress triggered by glutamate [38]. Therefore, the prevention of MAPK phosphorylation is crucial for defining the protective effects of neuroprotectants against glutamate toxicity. The present study, subsequently, examined whether compound **1** (50 µM), which is a potent neuroprotective compound of *R. elliptica* seed extract, prevents the phosphorylation of MAPKs increased by glutamate-mediated oxidative stress. The results exhibit that the treatment of **1** reduced the phosphorylation of ERK, JNK, and p38 that was increased by using glutamate (Figure 5).

### 3.6. Anti-Apoptotic Effects of 1 in HT22 Cells

**Figure 6:**
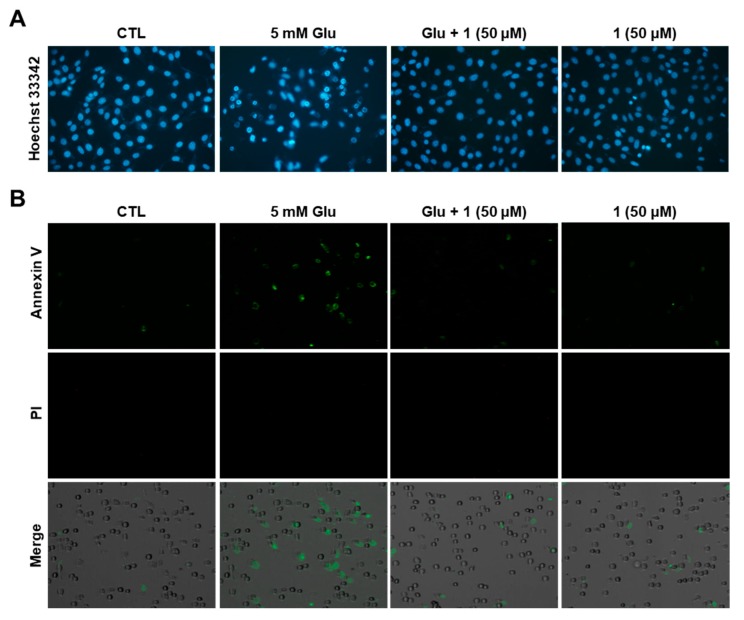
Compound **1** inhibited glutamate-induced apoptotic HT22 cell death. (**A**) HT22 cells were treated with 5 mM glutamate in the presence of **1** for 12 h and stained with Hoechst33342. Fluorescent images were obtained using a fluorescence microscope (40×). (**B**) HT22 cells were treated with 5 mM glutamate for 12 h in the presence of **1** and stained with Alexa Fluor 488-conjugated annexin V and propidium iodide. Fluorescent images were obtained using a Tali Image-Based Cytometer.

High concentrations of glutamate promote early necrotic and delayed apoptotic cell death in in vitro studies using cultured cerebellar granule and HT22 cells [35,36]. The present study mainly focused on the apoptotic pathway in HT22 cells. Chromatin condensation, shrinkage, and projection of phosphatidyl serine to the outer membrane are the most common characteristics of apoptotic cells [39,40]. To identify the anti-apoptotic effect of **1**, we conducted nuclear staining with Hoechst33342 to detect chromatin condensation. Chromatin condensation was markedly increased by glutamate. However, it was not observed in the compound **1** (50 µM)-treated groups (Figure 6A). Apoptotic cells were then analyzed using image-based cytometer after staining with Alexa Fluor 488-conjugated annexin V. Based on the representative fluorescent images, the number of annexin V-positive apoptotic cells increased due to the treatment with glutamate, whereas it was decreased in the 50 µM treatment of **1** (Figure 6B). Therefore, compound **1** possesses a potent anti-apoptotic property that prevents glutamate-induced oxidative stress.

## 4. Conclusions

We demonstrated that procyanidin B2 3″-*O*-gallate (**1**) can be a bioactive compound responsible for neuroprotective effects of *R. elliptica* seed extracts. The results suggest that procyanidin B2 3″-*O*-gallate (**1**), as a strong antioxidant, inhibited the accumulation of intracellular ROS and phosphorylation of MAPKs, which resulted in preventing apoptotic cell death induced by glutamate. Our findings revealed several underlying mechanisms of the protective effect of procyanidin B2 3″-*O*-gallate (**1**) and its potential for therapeutic use against glutamate-related neurological disorders. Furthermore, this study supports that *R. elliptica* seeds can be a useful natural source applicable as functional foods against neurological disorders. In order to clarify our conclusions, however, further studies are needed to identify the upstream factors regulated by procyanidin B2 3″-*O*-gallate as well as to verify those effects in animal models of neurological diseases.

## Supplementary Materials

The following are available online at https://www.mdpi.com/2218-273X/9/9/412/s1, Figure S1: LC/MS analysis of the MeOH extract, Table S1: ^1^H and ^13^C NMR data of compound **1** and the reported value of **1**, Figure S2: The ^1^H NMR spectrum of **1**, Figure S3: The ^13^C NMR spectrum of **1**. General experimental procedures, extraction and isolation, NMR data and quantitative analysis of procyanidin B2 3″-*O*-gallate, the UV chromatogram of LC/MS analysis, the cell culture, determination of cell viability, and the intracellular ROS assay are available free of charge on the Internet.
