# Supplementary material for: Procyanidin B2 3″-O-gallate Isolated from Reynoutria elliptica Prevents Glutamate-Induced HT22 Cell Death by Blocking the Accumulation of Intracellular Reactive Oxygen Species
Source: Biomolecules. 2019 Aug 26;9(9):412. doi: 10.3390/biom9090412 (PMC6769555; doi:10.3390/biom9090412)
Supplement: Supplementary file 1 [file biomolecules-09-00412-s001.pdf]

biomolecules

Communication

Procyanidin B2 3′′-*O*-gallate Isolated from *Reynoutria elliptica* Prevents Glutamate-Induced HT22 Cell Death by Blocking the Accumulation of Intracellular Reactive Oxygen Species

Ji Hoon Song [1,†], Sil Kim [2,†], Jae Sik Yu [2], Do Hwi Park [3], Song-Yi Kim [4], Ki Sung Kang [3], Sullim Lee [5,*] and Ki Hyun Kim [2,*]

[1] Jeju Institute of Korean Medicine, Jeju 63309, Republic of Korea
[2] School of Pharmacy, Sungkyunkwan University, Suwon 16419, Republic of Korea
[3] College of Korean Medicine, Gachon University, Seongnam 13120, Republic of Korea
[4] Department of Anatomy and Acupoint, College of Korean Medicine, Gachon University, Seongnam 13120, Republic of Korea
[5] Department of Life Science, College of Bio-Nano Technology, Gachon University, Seongnam, 13120, Republic of Korea
[*] Correspondence: sullimlee@gachon.ac.kr; Tel.: +82-31-750-8573 (S.L.) and khkim83@skku.edu; Tel.: +82-31-290-7700 (K.H.K.)
[†] These authors contributed equally to this work.

Received: 10 July 2019; Accepted: 24 August 2019; Published: date

Supplementary Materials

Materials and Methods

General Experimental Procedures

A JASCO P-1020 polarimeter (JASCO, Easton, MD, USA) was utilized to record optical rotation data. UV spectra were acquired on an Agilent 8453 UV-visible spectrophotometer (Agilent Technologies, Santa Clara, CA, USA). The NMR studies were performed using a Bruker AVANCE III 850 NMR spectrometer operating at 850 MHz (^{1}H) and 212.5 MHz (^{13}C). Preparative high-performance liquid chromatography (HPLC) was performed with a Waters 1525 binary HPLC pump equipped with a Waters 996 photodiode array detector (Waters Corporation, Milford, CT, USA) and column temperature was maintained at 30 °C. Semi-preparative HPLC was conducted with a Shimadzu Prominence HPLC system with SPD-20A/20AV Series Prominence HPLC UV-Vis detectors (Shimadzu, Tokyo, Japan) and column temperature was maintained at 30 °C. LC/MS analysis was performed on an Agilent 1200 Series HPLC system (Agilent Technologies, Santa Clara, CA, USA) equipped with a diode array detector and a 6130 Series ESI mass spectrometer with an analytical Kinetex (4.6 × 100 mm, 3.5 μm) HPLC column. The mobile phase consisted of H_2O (A) and MeOH (B) with a gradient system as follows: 10–100% B (0–30 min); 100 % B (30–40 min); 100-10% B (40–41 min); 10% B (41–50 min). The flow rate of the mobile phase was 0.3 mL/min, and the column temperature was maintained at 40 °C. A 5 μL aliquot from 100 μL of samples (0.01 mg/μL) was used in the positive-mode ESIMS at *m/z* 100-1000 Da range with acquisition times of 0.2 s in the centroid mode. The ESI conditions were set as follows: capillary voltage 2.0 kV, convoltage 50 V, source temperature 120 °C, desolvation temperature 350 °C, and desolvation gas flow 800 L/h. High purity nitrogen gas was used as the nebulizer and auxiliary gas. The collision energy for the detection of the precursor ions was set to 3 eV. RP-C_{18} silica gel (Merck, 40–63 μm) was used for column chromatography. Precoated silica gel F_{254} plates and RP-18 F_{254s} plates (Merck, Darmstadt, Germany)

were used for thin-layer chromatography (TLC). Spots on TLC were detected under UV light or by heating after spraying with anisaldehyde-sulfuric acid.

Extraction and Isolation

Dried seeds of *R. elliptica* (110 g) were extracted three times with 99.9% MeOH (200 mL × 3 days) at room temperature and then the extracts were filtered through Whatman filter paper no. 2 (pore size: 8 μm). The filtrates were combined and concentrated *in vacuo*, thus yielding crude MeOH extract (8.3 g). A stock solution of the MeOH extract was prepared at 25 mg/mL in DMSO and evaluated for its neuroprotective activity on glutamate-induced excitotoxicity in HT22 cells. The MeOH extract was dissolved in sterile distilled water, and a small aliquot of the MeOH extract was sequentially injected into the LC/MS and eluted with a gradient solvent system of MeOH–H_2O (1:9–9:1, flow rate: 0.3 mL/min, UV 254 nm), which revealed the presence of procyanidin dimer gallate with a molecular ion peak at *m/z* 731 $[M + H]^+$ in positive ESI mode by comparison with our house-built UV library in LC/MS. The MeOH extract (8.0 g) was separated by preparative reverse-phase HPLC using an Agilent Eclipse C18 column (21.2 × 250 mm; flow rate: 5 mL/min) with MeOH–water (1:9–9:1, gradient system) to yield five fractions (F1–F5). All the fractions were subjected to LC/MS and eluted with a gradient solvent system of MeOH–H_2O (1:9–9:1, flow rate: 0.3 mL/min, UV 254 nm) to identify the target constituent, procyanidin dimer gallate. Based on the LC/MS data, one major peak of procyanidin dimer gallate was detected in the F2 fraction. The F2 fraction (0.9 g) was further separated by reverse-phase semi-preparative HPLC on a Phenomenex Luna column (C18, 250 × 10.0 mm, 5 μm, flow rate: 2 mL min^{-1}) with 50% MeOH, thereby generating procyanidin B2 3″-*O*-gallate (**1**) (23.0 mg). Compound **1** was dissolved in DMSO at a concentration of 50 mM and their protective effect and underlying protective mechanism examined against glutamate-induced HT22 cell death. The purity of compound **1** used for the biological studies was determined to be >95% by LC/MS analysis.

Procyanidin B2 3′′-O-gallate (1)

Red and violet solid; $[\alpha]_D^{25}$ -42.0 (Me_2CO); UV (MeOH) λ_{max} 205, 215, 280 nm; ^{1}H (850 MHz) and ^{13}C NMR (212.5 MHz), see Table 1; ESI-MS (*m/z*) 731 $[M + H]^+$.

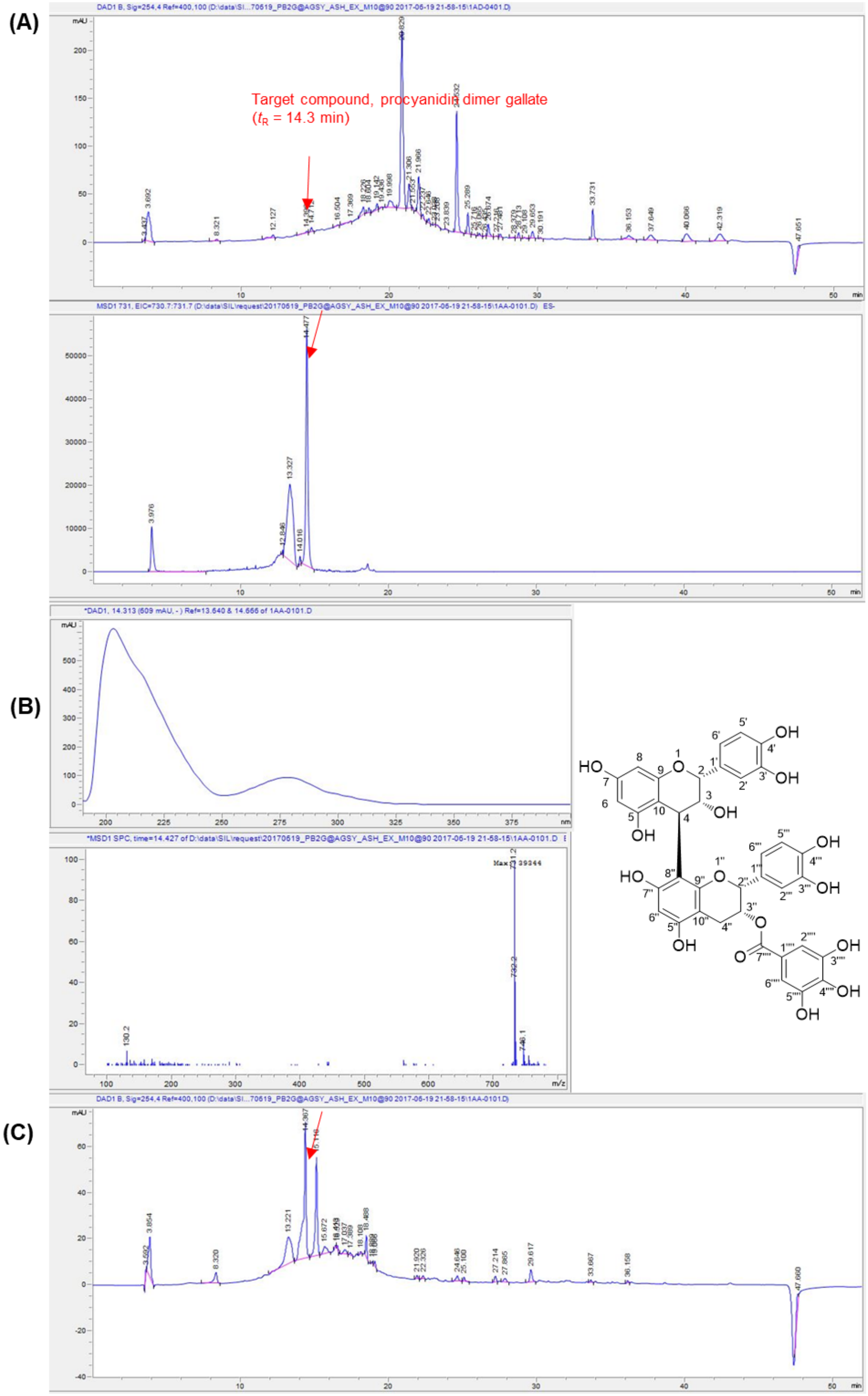

Figure S1. (A) UV chromatogram of LC/MS analysis of the MeOH extract (detection wavelength was set at 254 nm) and extracted ion chromatogram (EIC) for *m*/*z* 731 [M + H]$^+$ in positive ESI mode. (B) UV spectrum of the peak at retention time 14.3 min, positive ion-mode ESI-MS data of the peak, and the chemical structure of procyanidin B2 3″-*O*-gallate. (C) UV chromatogram of LC/MS analysis of the F2 fraction (detection wavelength was set at 254 nm), which indicated the presence of the peak of procyanidin dimer gallate.

Table S1. ^{1}H and ^{13}C NMR data of compound **1** and the reported value of **1**.[a]

Position	**1**[b]		**1** (reported one)[c] [1]	
	δ_C[b]	δ_H (*J* in Hz)	δ_C[b]	δ_H (*J* in Hz)
2	75.9 d	5.34 br s	75.8 d	5.31 br s
3	71.1 d	4.05 br s	71.7 d	4.00 br s
4	35.4 d	4.25 br s	36.0 d	4.45 br s
5	154.9 s		154.3 s	
6	95.3 d	5.65 d (2.0)	95.4 d	5.79 d (2.0)
7	154.0 s		153.4 s	
8	95.3 d	5.55 d (2.0)	95.4 d	5.54 d (2.0)
9	155.1 s		155.5 s	
10	106.6 s		103.3 s	
1'	130.2 s		131.2 s	
2'	113.7 d	7.08 d (2.0)	114.7 d	6.96 d (2.0)
3'	144.6 s		143.6 s	
4'	144.8 s		144.1 s	
5'	117.9 d	6.84 d (8.0)	116.1 d	6.81 d (8.0)
6'	119.2 d	7.05 dd (2.0, 8.0)	119.5 d	6.72 dd (2.0, 8.0)
2''	78.1 d	4.67 br s	77.7 d	4.47 br s
3''	68.8 d	5.21 d (5.0)	69.6 d	5.25 d (4.5)
4''	26.5 t	2.88 d (17.0); 3.05 dd (5.0, 17.0)	26.2 t	2.87 d (16.0); 2.96 dd (4.5, 16.0)
5''	154.9 s		154.4 s	
6''	95.3 d	6.14 s	96.1 d	6.23 s
7''	154.3 s		153.8 s	
8''	108.7 s		108.2 s	
9''	154.9 s		154.9 s	
10''	98.5 s		99.8 s	
1'''	129.3 s		130.1 s	
2'''	114.6 d	6.68 d (2.0)	115.0 d	6.63 d (2.0)
3'''	144.5 s		143.2 s	
4'''	144.8 s		143.9 s	
5'''	114.7 d	6.74 d (8.0)	115.5 d	6.69 d (8.0)
6'''	120.3 d	6.71 dd (2.0, 8.0)	119.7 d	6.46 dd (2.0, 8.0)
1''''	138.4 s		138.2 s	
2''''	108.9 d	6.94 s	109.8 d	6.87 s
3''''	145.0 s		144.7 s	
4''''	145.1 s		144.7 s	
5''''	145.0 s		144.7 s	
6''''	108.9 d	6.94 s	109.8 d	6.87 s
7''''	166.3 s		167.5 s	

[a] Coupling constants (in parentheses) are in Hz. [b] NMR data were measured at 850 MHz (^{1}H) and 212.5 MHz (^{13}C) in 10% DMSO in CD_3OD. [c] NMR data were measured at 400 MHz (^{1}H) and 100 MHz (^{13}C) in 10% D_2O in H_2O. Ref. [1] Tarascou, I.; Barathieu, K.; Simon, C.; Ducasse, M.A.; Andre, Y.; Fouquet, E.; Dufourc, E.J.; de Freitas, V.; Lqguerre, M.; Pianet, I. A 3D structural and conformational study of procyanidin dimers in water and hydro-alcoholic media as viewed by NMR and molecular modeling. *Magn. Reson. Chem.* **2006**, *44*, 868-880.

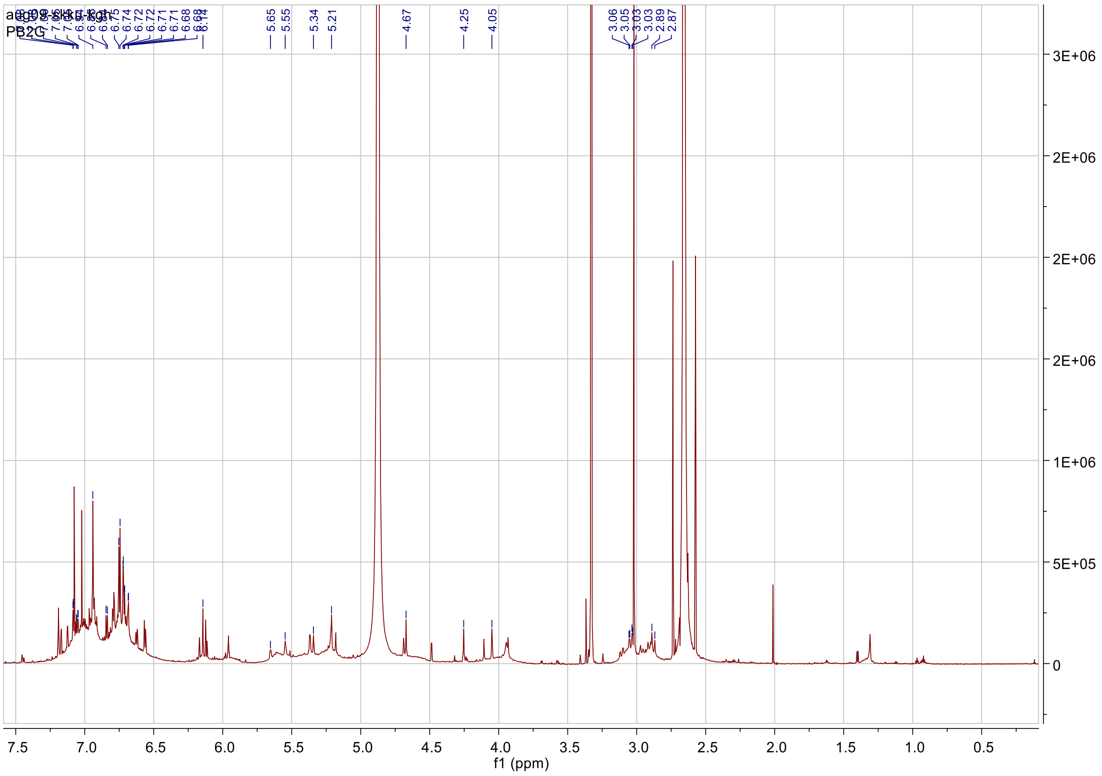

Figure 2. The ^{1}H NMR spectrum of **1** (850 MHz).

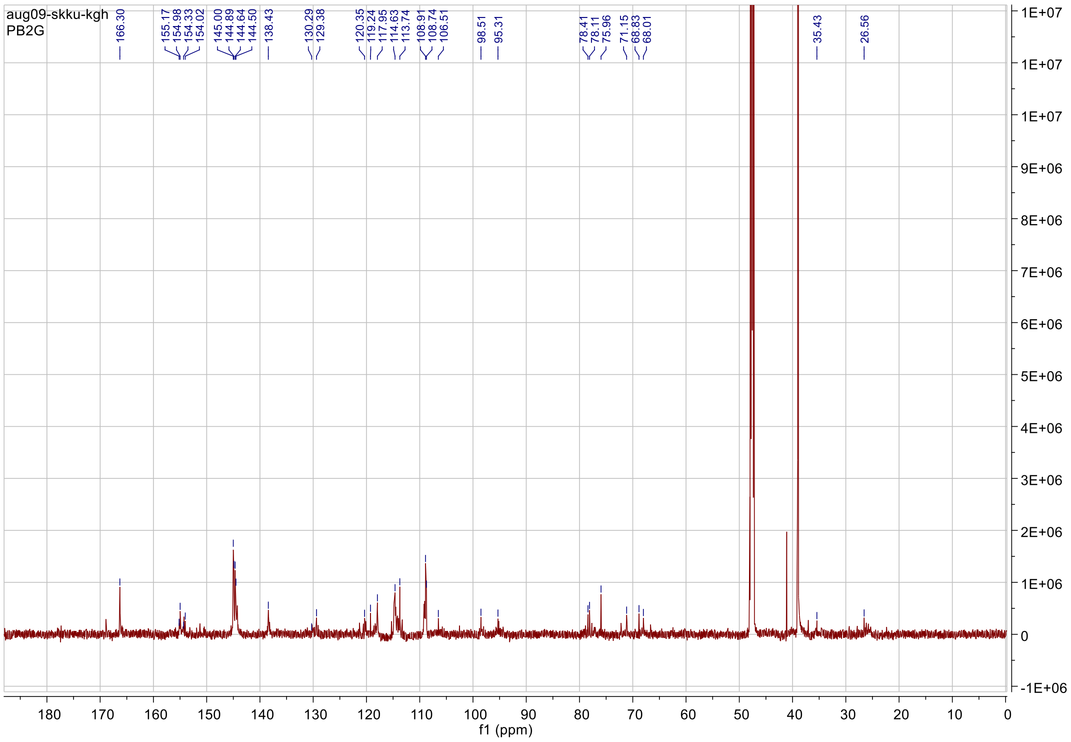

Figure 3. The ^{13}C NMR spectrum of **1** (212.5 MHz).

Quantitative Analysis of Procyanidin B2 3′′-O-gallate

The detection of **1** was analyzed using an LC-MS, Agilent 1200 Series analytical system equipped with a photodiode array detector combined with a 6130 Series ESI mass spectrometer. Calibration curves and linear regression equations were generated for the external standard, compound **1**. Compound **1** (1.0 mg) was dissolved in MeOH (1.0 mL), and the standard stock solution was further diluted with MeOH to provide solutions of 0.6, 1.5, 2.0, 3.0, and 5.0 μg/mL. The crude MeOH extract (1.0 mg) of *R. elliptica* seeds was dissolved in MeOH (1.0 mL), generating the sample stock solution, which was further diluted with MeOH to provide a solution of 100 μg/mL. Each solution was filtered through a 0.45 mm hydrophobic polytetrafluoroethylene filter and finally analyzed by LC/MS using a Kinetex C18 column (2.1 × 100 mm, 5 μm; Phenomenex, Torrance, CA, USA) set at 25 °C. The mobile phase consisting of formic acid in water [0.1% (*v*/*v*)] (A) and methanol (B) was delivered at a flow rate of 0.3 mL/min by applying the following programmed gradient elution: 10%–90% (B) for 30 min, 100% (B) for 1 min, 100% (B) isocratic for 10 min, and 10% (B) isocratic for 10 min, to perform post-run reconditioning of the column. The injection volume was 15 μL. The quantification of **1** was based on the peak area obtained from the MS detection and calculated as equivalents of the standard. Content was expressed as micrograms per 1 mg of extract weight.

Cell Culture

For demonstrating the neuroprotective activity of MeOH extracts and compound **1**, the immortalized murine hippocampal HT22 cell line was grown on Dulbecco's Modified Eagle's medium (Corning, Manassas, VA, USA) supplemented with 10% fetal bovine serum and penicillin/streptomycin (Grand Island, NY, USA). The tissue culture plates were obtained from Falcon (BD Biosciences, Franklin Lakes, NJ, USA). Glutamate was obtained from Sigma-Aldrich (St. Louis, MO, USA). All other chemicals were of reagent grade.

Determination of Cell Viability

Cell viability was determined using a cell viability assay kit (EZ-CyTox; Daeil Lab Service, Seoul, Korea) according to the manufacturer's instructions. In brief, HT22 cells were plated onto 96-well plates at a density of 1×10^4 cells/well and allowed to attach for 24 h. Cells were then treated with 5 mM glutamate in the presence or absence of MeOH extracts or compound **1**. After exposure to glutamate for 24 h, the cells were incubated for an additional 30 min with 10 μL of EZ-CyTox reagent. The absorbance was measured at 450 nm using an E-Max microplate reader (Molecular Devices, Sunnyvale, CA). The results are represented as the percentage of cell viability compared to the control cells.

Intracellular ROS Assay

The levels of intracellular ROS were determined using 2′,7′-dichlorofluorescin diacetate (DCFDA; Sigma-Aldrich). HT22 cells were plated on black 96-well plates at a density of 1×10^4 cells per well. After exposure to 5 mM glutamate for 8 h, the cells were loaded with 10 μM DCFDA and incubated for 30 min. After washing cells thrice with phosphate buffered saline, the fluorescence intensity of DCFDA was measured using a fluorescent microplate reader (SPARK 10M; Tecan, Männedorf, Switzerland) at excitation and emission wavelengths of 495 and 517 nm, respectively. In addition, representative fluorescent images were obtained using a fluorescence microscope (IX50; Olympus, Tokyo, Japan) equipped with a charge-coupled device (CCD) camera.

© 2019 by the authors. Licensee MDPI, Basel, Switzerland. This article is an open access article distributed under the terms and conditions of the Creative Commons Attribution (CC BY) license (http://creativecommons.org/licenses/by/4.0/).
